# Extracellular matrix-derived materials for tissue engineering and regenerative medicine: A journey from isolation to characterization and application

**DOI:** 10.1016/j.bioactmat.2024.01.004

**Published:** 2024-01-17

**Authors:** Jennifer Noro, Helena Vilaça-Faria, Rui L. Reis, Rogério P. Pirraco

**Affiliations:** a 3B's Research Group, I3Bs – Research Institute on Biomaterials, Biodegradables and Biomimetics, University of Minho, Headquarters of the European Institute of Excellence on Tissue Engineering and Regenerative Medicine, AvePark, Parque de Ciência e Tecnologia, Zona Industrial da Gandra, 4805-017, Barco, Guimarães, Portugal; b ICVS/3B's – PT Government Associate Laboratory, Braga, Guimarães, Portugal

**Keywords:** Extracted ECM, Extraction methods, Characterization, ECM-derived applications

## Abstract

Biomaterial choice is an essential step during the development tissue engineering and regenerative medicine (TERM) applications. The selected biomaterial must present properties allowing the physiological-like recapitulation of several processes that lead to the reestablishment of homeostatic tissue or organ function. Biomaterials derived from the extracellular matrix (ECM) present many such properties and their use in the field has been steadily increasing. Considering this growing importance, it becomes imperative to provide a comprehensive overview of ECM biomaterials, encompassing their sourcing, processing, and integration into TERM applications. This review compiles the main strategies used to isolate and process ECM-derived biomaterials as well as different techniques used for its characterization, namely biochemical and chemical, physical, morphological, and biological. Lastly, some of their applications in the TERM field are explored and discussed.

## Highlights

• The main methods for ECM isolation from tissue, organs, and cells and subsequent decellularization are reviewed.
• Techniques for its biochemical, physical, morphological and biological characterization are described.
• Examples of the application of ECM-derived biomaterials are briefly compiled and discussed.
• Future focal points of research related with ECM-derived biomaterials are anticipated.

## Graphical abstract

**Figure:**
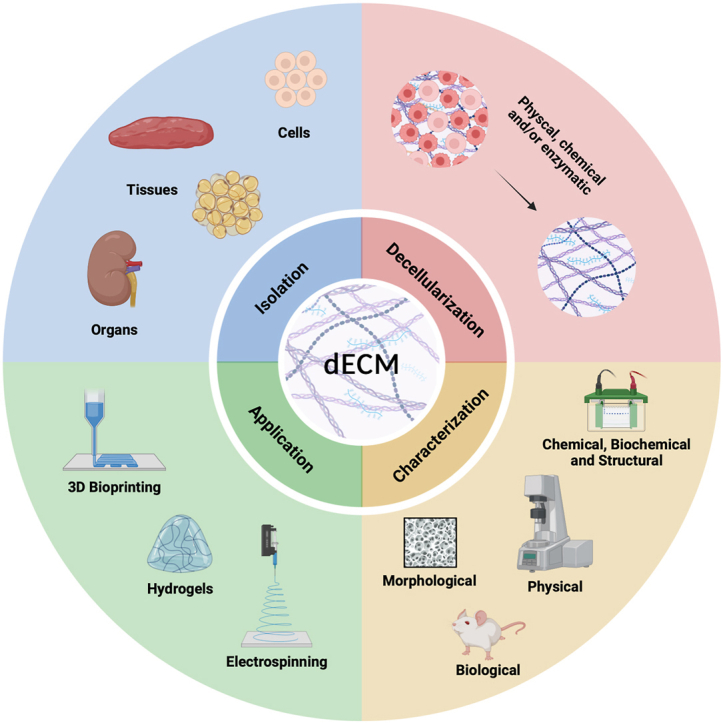

## 1 Introduction

Tissue Engineering and Regenerative Medicine (TERM) is an interdisciplinary field, that aims at finding solutions to repair or regenerate damaged tissues and organs. To achieve such solutions, an ECM surrogate such as a scaffold is often needed and biomaterials, from natural or synthetic origin, are used to obtain said surrogates [1]. The choice between natural and synthetic biomaterials represents a challenge due to the different properties of each subset of biomaterials. Scaffolds produced from synthetic materials afford easily tailorable mechanical properties at the cost of lower biocompatibility. Contrariwise, and in spite of the many recent advancements in the field, natural-based scaffolds, especially the ones based on ECM-derived proteins such as collagen, elastin, and keratin, offer good biocompatibility but present limitations in terms of mechanical tunability and stability. An ideal biomaterial would combine the best features of synthetic and naturally occurring materials knowing that in order to achieve a higher regeneration/repair potential, the biomaterial should provide a favorable microenvironment for cells [2]. Taking this into account, it's undeniable that a particular set of biomaterials has aroused greater interest during the last few years: extracellular matrix (ECM)-derived biomaterials. The ECM is a complex cell-produced 3D network that embeds cells in tissues and organs throughout the body, supporting their function and survival. Due to the ECM's intrinsic cyto and biocompatibility, its use to produce biomaterials has exponentially grown, with more than 600 original articles mentioning it published per year since 2020, according to WebOfScience. The typical path to obtain ECM-derived biomaterials involves firstly the depletion of potentially immunogenic materials such as DNA from the original material through a decellularization step. Characterization of decellularized ECM or of its components should follow to ensure the preservation of its biological activity. Depending of the desired application, the preservation of its original 3D structure may also be evaluated. These steps are in fact an oversimplification of the many ways proposed by researchers and engineers to obtain scaffolds using ECM-derived biomaterials. Given the growing importance of such biomaterials, an overview of the most relevant methodologies employed for their obtention becomes crucial.

**Fig. 1:**
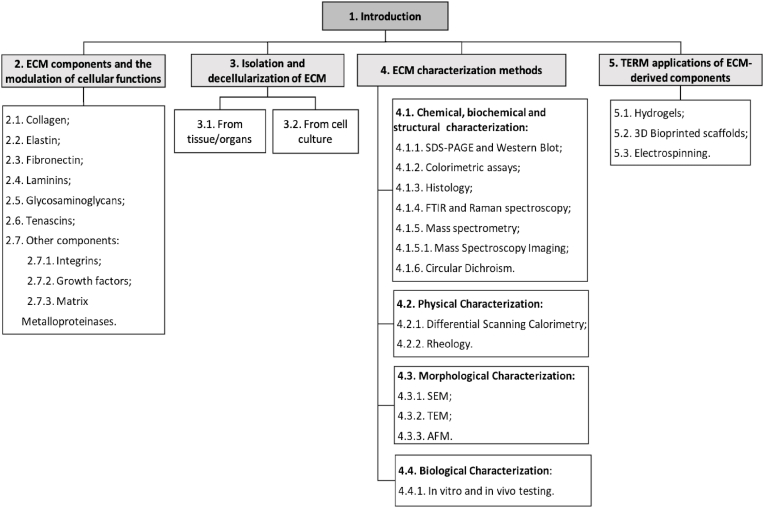
Overview diagram of the review.

In this context, the purpose of this paper is to review the main approaches to produce ECM-derived biomaterials, starting from the different sources (organs/tissues or cell culture), to the different decellularization processes (physical, chemical or enzymatic), types of characterization (biochemical, chemical, physical, morphological and biological), and finally, selected applications in the TERM field (hydrogels, bioprinting and electrospinning) (Fig. 1). It is important to emphasize that while it is possible to employ a fully decellularized organ/tissue and engineer it for TERM applications, the primary focus of this review is to provide an overview and discussion of examples from extracted ECM.

## 2 ECM components and the modulation of cellular functions

**Fig. 2:**
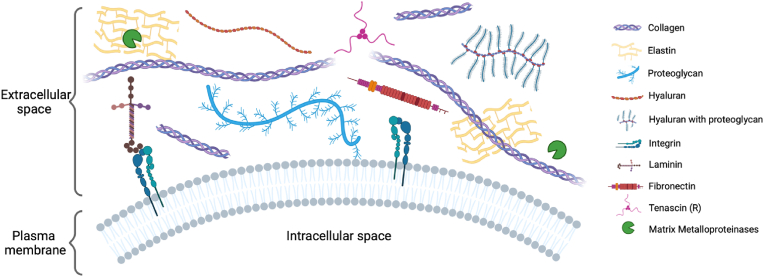
Representation of the extracellular matrix content. Created with BioRender.com.

The ECM is an organized 3D network produced by cells within the different tissues of the body, composed of several macromolecules whose final composition varies according to the tissue [3]. This network is essential for the cellular components’ survival since it functions not only as a physical scaffold but also has a fundamental role in several biochemical and biomechanical processes, influencing cellular activities and responses (e.g. proliferation, differentiation, migration, and apoptosis) required for tissue homeostasis [4]. Although the ECM of different tissues is composed of the same key components, each tissue has a unique composition and structure that differs according to the nature and function of the tissue [5]. Also, the ECM is a highly dynamic structure, being constantly changed by several post-translational modifications and remodeled during physiological and pathological conditions (e.g., cancer), which is why it is a key factor to consider for TERM applications. Although fundamentally composed of water and macromolecules, the ECM main components are proteins such as proteoglycans and fibrous proteins, which are central pieces for the support of the cellular constituents of tissues [6]. These proteins can be divided into two categories, as either structural (fibrous proteins) or non-structural proteins (proteoglycans and glycoproteins), depending on their role. Fibrous proteins are made up of polypeptide chains with a sheet-like structure and include collagens and elastins [7,8]. On the other hand, non-fibrous proteins are composed of glycosaminoglycans (GAGs) chains linked to a specific protein core and include fibronectins, tenascins, and laminins [9]. Other essential components of the ECM include growth factors (GFs), integrins, and a variety of matrix metalloproteinases (MMPs) (Fig. 2).

### 2.1 Collagen

Collagen is the most abundant fibrous protein in the ECM. In fact, it is the most abundant protein within the connective tissues (e.g., tendon and skin) of the human body, with around 30 identified subtypes [10]. This molecule is involved in several mechanisms such as paracrine regulation of the cell behavior, promotion of adhesion, proliferation, and differentiation [11,12]. Collagen is involved in such mechanisms by aiding in the sequestration of GFs and other signaling molecules involved in paracrine regulation, which activates several cascades of signaling pathways that promote the above-mentioned processes. Mechanotransduction is also regulated by changes in collagen stiffness and the direct force applied to the cells, affecting several cell signaling pathways [13].

The presence of binding domains on other molecules that allow a close association with other proteins, allowing water uptake for tissue hydration and the absorption of forces applied, therefore influencing cell behavior and ECM physical properties [14,15]. These properties depend on the tissue and although collagen fibers are usually a heterogeneous mix of different types, usually one collagen form predominates. Type I collagen is the dominant form found in almost all tissues, such as skin, tendons and bone. Type II collagen is found more on cartilage and cornea, while type III collagen is widely found in blood vessels’ walls. Overall, collagen biochemical and biomechanical properties dictate the tissue-specific ECM function and healthy phenotype, since it has already been reported that alterations/mutations in collagen are implicated in various clinical pathologies such as arterial aneurysms, epidermolysis bullosa acquisita, osteogenesis imperfecta, as well as tumorigenesis, among other diseases [5,16].

### 2.2 Elastin

Elastin is another major ECM fiber, composed of tropoelastin subunits that are cross-linked with a layer of fibrillin microfibrils, which make up an elastic fiber [17]. This property allows elastin-rich tissues to possess elasticity and permits them to recover/recoil when subjected to repeated stretch [18,19]. This feature is important in tissues such as the lungs and blood vessels [20,21]. In the latter, elastin works closely with collagen [22]. In large arteries such as the aorta, elastin is responsible for the reversible extensibility during the stress-relaxation cycle, while collagen provides the strength and ability to withstand the high pressure in these large elastic arteries [23,24]. Indeed, some cardiovascular pathologies have been associated with elastin dysregulation in these tissues over time, such as calcific aortic valve disease, and congenital supravalvular aortic stenosis [23,25]. Due to all this, elastin is widely used in tissue engineering as a biomaterial [26].

### 2.3 Fibronectin

Fibronectin is expressed on the basement membrane of a variety of cell types that have essential functions during vertebrates development [27]. This protein is composed of two subunits produced in the form of a disulfide covalently bonded dimer. These subunits can be broken down into 3 different types (type I, II, and III), in which each has different structures and some can undergo conformational changes, influencing cellular behavior, and therefore being implicated as an extracellular mechanoregulator [28]. *In vivo*, fibronectin is present in two different forms: cellular fibronectin, which is secreted by the mesenchymal cells, and plasma fibronectin, secreted by the hepatocytes. In the ECM, once fibronectin is secreted, it interacts with integrins. This interaction triggers a conformational change in fibronectin, starting a process of self-association, leading to their assembly into fibrils. The interaction between fibronectin and integrins is fundamental for optimal ECM formation [29,30], which in turn is needed for cell adhesion and migration, influencing a myriad of processes such as the wound healing response to injury [6] and vascular morphogenesis [31]. Fibronectin has also been implicated in cardiovascular diseases [32,33], and tumor progression [34].

### 2.4 Laminins

The laminin family is composed of multidomain, heterotrimeric glycoproteins that are found in basement membranes, crosslinked with other ECM components. Each of these trimeric isoforms consist of 3 chains (α, β, and γ) [35], which exist in genetically distinct forms, depending on their cell and tissue specificity [36]. In fact, in complex vertebrates, 16 trimeric isoforms have been identified [37]. Laminin actively influences cell behavior, since it has an effect on cell adhesion, differentiation, and migration, and like fibronectin, also contributes to ECM structure [6]. It also supports phenotype stability, and resistance against apoptosis by interacting with cell membrane receptors such as integrins and dystroglycan. In fact, laminins are the first ECM component appearing in early embryonic development, and have a crucial role in organogenesis [38]. They contribute to the homeostasis of numerous tissues such as the blood vessels, kidneys, connective tissues like cornea, and are required for tendons proper healing [39]. Furthermore, these proteins have been implicated in angiogenesis, contributing to vessel growth and maturation [40]. Also, mutations in laminin chains are associated with human congenital disorders, such as Pierson syndrome (β2 chain mutation) [41], congenital muscular dystrophy (α2 chain mutation) [42], and junctional epidermolysis bullosa (α3, β3, and γ2 chains mutation) [43].

### 2.5 Glycosaminoglycans

Glycosaminoglycans (GAGs) are a group of linear polysaccharides that are composed of repeating units of disaccharides [44]. Typically, these disaccharides consist of either *N*-acetylglucosamine or *N*-acetylgalactosamine linked to a residue of hexauronic or galactose [45]. Despite the simplicity of the sugar components, the structural analysis of GAGs is challenging due to their intricate modification patterns, including epimerization and sulfation [46]. One of the distinguishing features of GAGs is their highly negative charge, which is attributed to the presence of sulfate and/or carboxylic groups within their structure [47].

Due to their remarkable structural diversity, GAGs possess the ability to interact with a broad spectrum of biological molecules. These interactions play crucial roles in various biological processes, including cell migration, division, angiogenesis, and collagen fibrillogenesis [48]. Additionally, GAGs exhibit therapeutic potential in addressing pathologic conditions such as thrombosis, neovascularization, cancer, and inflammation, making them highly desirable for therapeutic applications [44].

There are four main classes of glycosaminoglycans: hyaluronan, chondroitin sulfate/dermatan sulfate, heparan sulfate/heparin, and keratan sulfate [44]. These GAGs exhibit a wide range of biological functions, some of which are briefly described next. Heparin functions as an anticoagulant [49] and plays a role in regulating inflammation [50]. Heparan sulfate interacts with cytokines, chemokines, and interleukins, contributing to various cellular processes [51,52]. Chondroitin sulfate helps prevent inflammation, modulates the immune response, and regulates cell adhesion to the ECM [53,54]. Dermatan sulfate is involved in collagen organization [55] and the regulation of cell-matrix interactions [56]. Keratan sulfate is responsible for tissue hydration [57], and hyaluronan contributes to stabilizing connective tissue and organizing the ECM [58].

### 2.6 Tenascins

Tenascins are a multifunctional group of ECM glycoproteins, with tenascin-C being the first described and best studied. Other tenascin family members are tenascin-R, -W, -X, and -Y [59]. Tenascins have a characteristic modular structure composed of identical subunits formed from variable numbers of repetitive domains, which include heptad repeats, a series of fibronectin type III domains, epidermal growth factor (EGF)-like repeats, and a C-terminal globular domain [60].

Tenascin-C is highly expressed during embryonic development at sites of branching morphogenesis, and in the developing organs such as kidney, mammary glands, and teeth, while is also present in sites of epithelial-mesenchymal interactions, as well as in the developing smooth muscle, cartilage, and bone. In the adult life, this tenascin is only expressed at high levels in tendons, and in some stem cell niches, while only transiently elevated when tissue injury occurs and is later down-regulated [61]. One interesting fact about this tenascin is that it shares a structural relationship with fibronectin, limiting the fibronectin-mediated cell spreading when both proteins are combined since tenascin-C differs in adhesive function [62]. In fact, overexpression of tenascin-C leads to a series of malfunctions and it has been found elevated in rheumatoid arthritis and in several pathological cardiac conditions [63,64].

Tenascin-R is a more locally focused protein, mainly found in the central nervous system (CNS), where it is mainly expressed around certain glial cells and perineural networks of the developing and adult CNS [65].

Tenascin-W has the potential to act as an adhesion modulatory protein and has been associated with osteogenesis since it is, during development, highly expressed during bone and smooth muscle morphogenesis and palate formation, while in the adult it is mainly found in certain stem cell niches and in organs such as the kidneys. In fact, when present in osteoblasts culture, this tenascin is able to promote bone development, angiogenesis, cell adhesion and migration [66]. Apart from the above-mentioned cases, tenascin-W is usually missing from the ECM of most organs, although being vastly found in many solid tumors, which could turn it into a potential tumor marker and target [67].

Tenascin-X, a less glycosylated form than tenascin-C and –R, is present in almost all tissues, being highly expressed in the heart, skeletal muscle, tendon, and skin [68]. This tenascin is important in the organization and maintenance of the ECM structure and stability, since it regulates the formation of collagen fibrils in the matrix. Insufficiency of this protein may lead to clinical manifestations similar to Ehlers-Danlos syndrome hypermobility type and benign joint hypermobility syndrome [69]. Finally, tenascin-Y is the avian equivalent to tenascin-X [70].

### 2.7 Other components

#### 2.7.1 Integrins

Integrins are important cell adhesion mediators since they work as a link between the cellular cytoskeleton and the ECM [71]. Integrins are heterodimeric transmembrane receptors formed through a noncovalent association between two transmembrane glycoproteins, the α and β subunits. In mammals, 18 different types of α subunits have been discovered, as well as 8 different β subunits, which together can form 24 different combinations, each with different specificity regarding both tissue and ECM [72]. Through the interaction of integrins with ECM proteins, several signaling cascades are activated, mediating cell migration, proliferation, differentiation, and survival [73]. Indeed, integrins are able to mediate stable adhesion to basement membranes though the interaction with collagens and laminins, mediate matrix assembly and motility, while also being able to interact with the immune system since they can bind to bacterial polysaccharides and viral coat proteins [74,75]. In fact, integrin activation can occur in a bidirectional manner, where they can signal information from the inside to the outside of the cell and vice-versa, providing a constant flow of information between intra and extracellular compartments [76]. However, this bidirectional signaling is also critical for cancer onset and development, where several studies have correlated the high expression of certain integrins with the progression of several tumors [77,78].

#### 2.7.2 Growth factors

The ECM has the unique feature of acting as a reservoir of bioactive molecules such as GFs and cytokines. GFs such as the vascular endothelial growth factor, fibroblast growth factor, and transforming growth factor-β (TGF-β) are linked to the ECM either through heparan or heparan sulfate residues and are activated by several processes such as wound healing and tissue remodeling, becoming fundamental for the correct development and differentiation of many tissues [79]. In fact, the ECM contributes to ligand maturation, since TGF-β, for example, is stored in the ECM in its latent form, remaining inactive until activated by MMPs [80]. In addition, it can also establish concentration gradients, with a temporal and spatial regulation of their bioavailability. This occurs due to the presence of binding sites for growth factors on ECM proteins, allowing a concentration gradient near their cell surface receptors, which is vital in processes such as patterning in developmental stages [12,81]. Indeed, this establishes ECM as an organizing complex of the cell signaling dynamics.

#### 2.7.3 Matrix metalloproteinases

MMPs are an important group of zinc-dependent endopeptidases with a central role in ECM remodeling [82]. The ECM is constantly changing, its components are always being produced and deposited, degraded, or altered. In this process, MMPs are the key group of enzymes involved in this process. Dozens of MMPs have been identified, and they can be classified in 6 distinct groups (collagenases, gelatinases, stromelysins, matrilysins, membrane type-MMPs, and other MMPs) depending on their substrate specificity and structural features [83]. Since MMPs overlap in substrate specificity, they present a high ability to breakdown the matrix, and as such, require a tight regulation. This mediated proteolysis is regulated at the mRNA level, by their preservation at a quiescent state prior to their activation, and, lastly, with the presence of specific tissue inhibitors of MMPs to counteract damage to the matrix [84,85]. MMPs play an extremely important role as modulators of cellular dynamics and interactions, in the response to environmental changes that promote normal/pathological development [86], inflammation [87], and even as targets for anti-cancerous therapies [88].

## 3 Isolation and decellularization of ECM

### 3.1 From tissue/organs

As previously mentioned, one of the approaches of the TERM field for functional tissue repair is centered on the use of scaffolds made from biomaterials. These biomaterials can be categorized as synthetic or as naturally occurring if collected from biological sources [89]. Synthetic materials are usually composed of manufactured substrates such as polymers, metals, and/or chemicals, which allow high production precision, with well-defined material properties, that contributes to minimal batch-to-batch variability, and in turn, an expected tissue/host response [90]. However, this type of material can trigger a foreign body reaction, being linked with a pro-inflammatory and fibrotic host effect [91]. In contrast, natural materials originate from natural occurring sources such as animals, plants, algae and microorganisms. ECM-derived biomaterials are an example of natural origin biomaterials. These materials can comprise the whole ECM extract or purified ECM components such as collagen type I, laminin, fibronectin, or hyaluronic acid. ECM-derived biomaterials tend to have a more favorable host immune response since they are originated from a natural microenvironment, with the caveat of being less easy to control in terms of composition and mechanical properties [92,93]. Ideally, the perfect biomaterial would combine the precision and control of the synthetic materials manufacturing with the biologically favorable properties of ECM-derived materials in order to promote beneficial tissue remodeling [94]. As such, in the past few decades there has been an increased interest in the production of scaffolds based on ECM-derived materials that could fill this gap. ECM scaffolds are normally prepared by the decellularization of source tissues or organs, either xenogeneic or allogeneic in origin, from a great variety of available anatomical sites [95,96]. The main goal of the decellularization process is the removal of all cells and genetic material present on the native tissue, in order to avoid initiating an immune response when applied. Depending on the desired application, the decellularization process should be carefully selected. If the goal is to use directly the ECM as scaffold, the decellularization process should preserve the composition and 3D ultrastructure of the native ECM. If the purpose is to extract ECM to be used as a raw material, the preservation of its 3D structure is not a concern.

**Fig. 3:**
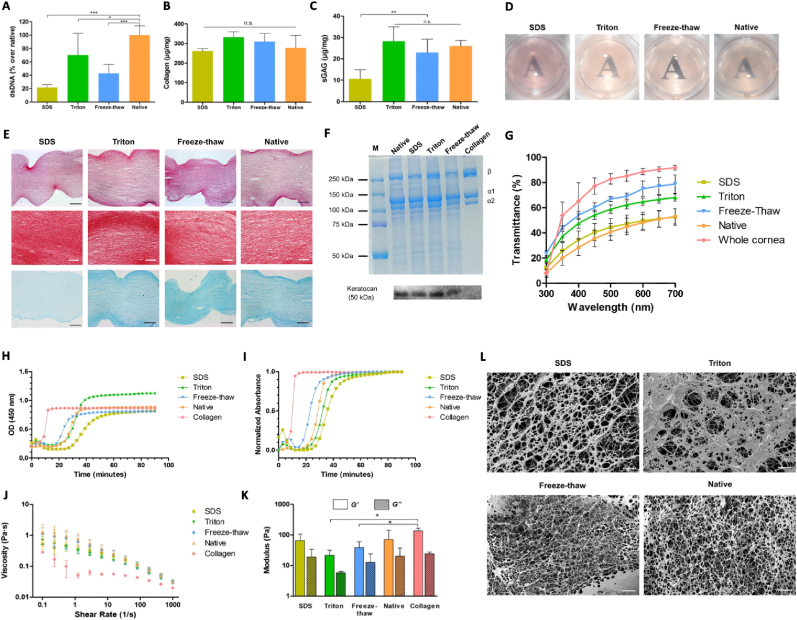
The impact of different decellularization methods (freeze-thaw, SDS and Triton) on ECM-derived hydrogels obtained from porcine corneas; (A), (B), (C) DNA, collagen and sGAG content, respectively; (D) transparency; (E) histological examination, stained with haematoxylin and eosin, picrosirius red and Alcian blue; black scale bar = 100 μm, white scale bar = 50 μm; (F) biochemical composition via SDS-PAGE, and Western blot against keratocan; (G) transmittance; (H) raw values of gelation kinetics via turbidimetric analysis; (I) normalized data of gelation kinetics via turbidimetric analysis; (J) viscosity measurements at increasing shear rates by rheology analysis; (K) storage modulus (G′) and loss modulus (G″) by rheology analysis; (L) cryoSEM micrographs at 1000x, scale bar = 10 μm [116]. Copyright 2019, Springer Nature.

The resulting material can be used as a template that provides signaling molecules important for functional tissue repair. Indeed, several studies have demonstrated that ECM-derived scaffolds influence cell viability and proliferation [97], promote cell differentiation [98], and have shown promising results for tissue repair in damaged Achilles tendon [99], in skin wound healing [100], and even in spinal cord injury [101]. In fact, a recent study has shown that ECM-derived hydrogels could be an optimal substitute for Matrigel, when it comes to the culture of gastrointestinal organoids [102]. The physical and biochemical properties of the ECM scaffold significantly depend on a plethora of factors that need to be considered beforehand, such as the tissue source, the chosen method of decellularization, and the processing steps taken after decellularization (e.g., terminal sterilization, and chemical crosslinking). Accordingly, there are several protocols commonly used for tissue decellularization, already described for almost every tissue in the body [103]. In general, most approaches use a combination of physical, chemical and enzymatic methods (Table 1). Physical or mechanical methods (e.g., freeze-thaw cycles, sonication, and high hydrostatic pressure (HHP)) are commonly used and involve temperature and pressure procedures to disrupt cells and allow the release of cellular residues from the tissue of interest [[104], [105], [106]]. Chemical methods are based on the use of chemical agents such as surfactants (e.g., Triton X-100, sodium dodecyl sulfate (SDS), and 3-((3-cholamidopropyl) dimethylammonio)-1-propanesulfonate (CHAPS)), acid/bases (e.g., peracetic acid, and sodium hydroxide (NaOH)), and solvents (e.g., acetone, and methanol) for the disruption of the lipid cell membrane, culminating with cell lysis and removal of cytosolic and genomic material [[107], [108], [109], [110]]. However, this type of decellularization method tends to be more damaging to the ECM native structure and integrity when compared to the physical protocols, and need extra washing steps due to the inherent cytotoxicity of the reagents used. Finally, enzymes are also used in combination with other approaches in order to assist decellularization, providing high precision in the elimination of specific molecules. Proteases (e.g., trypsin, pepsin, and dispase) are used to aid decellularization, since they are able to break cell-matrix interactions, through hydrolysis of amide bonds. Endo and exonucleases (e.g., DNAse, and RNAse) are very useful for the elimination of nuclear waste after cell lysis induced by other decellularization agents [[111], [112], [113]]. Since the presence of genetic material in biologic scaffolds can induce a pro-inflammatory response when implanted *in vivo*, leading to functional failure, several criteria have been defined in order to confirm decellularization efficiency and reduced immunogenicity of the ECM-derived scaffolds. These general guidelines include the following: Haematoxylin & Eosin (H&E) and 4′,6-diamidino-2-phenylindole stainings to verify cell and nuclei absence, and quantitative measurement of the remaining DNA content, which should not surpass 50 ng of double stranded DNA per mg of dry weight ECM and 200 base pair in fragment length [114]. Additional characterization should be performed in order to assess the remaining protein content of the ECM, highlighting the structural proteins such as collagen, fibronectin, and laminin, as well as glycosaminoglycans (GAGs), and GFs. Furthermore, if the ECM is to be used directly as a scaffold, the mechanical/elastic properties including elastic modulus and tensile strength should be assessed and evaluated depending on the final application [115]. Fig. 3 depicts a comparison between the effect of physical (freeze-thaw) and chemical (SDS and Triton) decellularization processes on the mechanical and physicochemical properties of the isolated ECM [116]. The authors observed that all decellularization methods significantly reduced the DNA content while preserving all major ECM components (as assessed by colorimetric assays, histological analysis, and SDS-PAGE). In terms of biocompatibility, SDS rendered cytotoxic hydrogels, while all other methods afforded good cytocompatibility. The hydrogels produced after freeze-thaw decellularization were demonstrated to be the most transparent and have the overall best properties.

**Table 1:** Summary of strategies used for tissue/organ decellularization.

Method	Agents and Techniques		Mechanism		Advantages	Disadvantages	References
**Physical**	Freeze-thaw		Intracellular ice crystals formation causes cellular membrane disruption		Simple and cost effective	Rapid freezing changes the ECM ultrastructure Incomplete decellularization	[104,106,124]
	Sonication		Sound waves aids the disruption and removal of cell debris		Complete disruption of the cell membrane	Excessive energy and sonication time can damage the ECM structure	[104,122,125]
	High Hydrostatic Pressure (HHP)		Creates high pressure in the tissue, inducing cell lysis		Easy to operate and fast	Denaturation or alteration of bioactivity/structure Expensive	[105,126]
**Chemical**	Surfactants	Ionic	SDS	Disruption of the lipid membrane	Effective removal of immunogenic components	Denaturation of ECM proteins Reduction in GF and GAGs content	[104,107,108,110,127,128]
		Non-Ionic	CHAPS				
		Zwitterionic	Triton X-100				
	Acid/Bases	Peracetic Acid; NaOH	Solubilization of cellular components and removal of cell debris		Sterilization effect Effective removal of nuclear and cytoplasmic components	Reduction in collagen, GF and GAGs content Disruption of collagen crosslinks (ECM microstructure) Decrease of mechanical properties	[108,129,130]
	Solvents	Acetone; Methanol	Dehydration, solubilization, and removal of lipids		Effective removal of lipidic content	Collagens damage Precipitation of proteins	[96,110,131]
**Enzymatic**	Trypsin		Breakdown of membrane peptides on the C-side of Arg and Lys		High specificity Effective removal of nuclear and cytoplasmic components	Prolonged exposure can disrupt collagen and elastin (ECM microstructure) Difficult to achieve complete decellularization	[111,127,132]
	Pepsin		Hydrolysis of amides from aromatic aminoacids		Broad specificity	Prolonged exposure can compromise ECM structural and biological activity	[133]
	Endo and Exonucleases	DNAse; RNAse	Degradation of nuclear content after cell lysis		Effective removal of DNA and RNA residues	Needs a previous cell lysis step	[112,129]

Although not the main focus of this review, it is worth noting that these decellularization techniques have also been explored in whole organs context, by using decellularization by perfusion, which leads to the conservation of the organ-derived 3D native ECM with an intact vascular tree [117]. This offers a route for the scaffold recellularization with site-specific cells and/or stem, and progenitor cells [118]. This method has opened new opportunities for the substitution of damaged/diseased organs since there are already reports suggesting the efficacy of kidney, liver, and pancreatic islet–derived matrices that sustain cellular viability [[119], [120], [121]].

In any case, the decellularization process unavoidably affects the ECM to some extent, and different decellularization agents have different effects on the resulting ECM biomaterials [116]. Depending on the protocol used, there could be detrimental effects on the GFs and GAGs present on the ECM, as well as damage to collagen and other ECM proteins. Therefore, it is of major importance to select the appropriate decellularization method, taking into account the several existing variables such as the tissue of interest, and its morphology, as well as the concentration, time, and sequence of the selected technique. For this reason, the chosen decellularization process needs to be adapted to the final aim and application, which requires a balance between the removal of the antigenic material (e.g., membrane lipids, cytosolic proteins, and nucleic acids), and the maintenance of the ECM integrity and functionality. More in-depth overviews of the different strategies to accomplish the decellularization of organs and tissues can be found elsewhere [96,122].

**Fig. 4:**
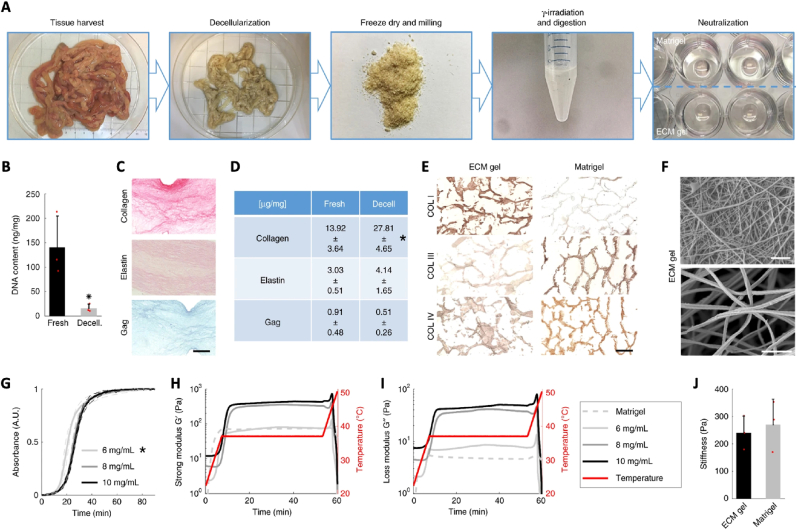
Extracellular matrix hydrogel isolation and characterization: (A) preparation of ECM from SI mucosa/submucosa; (B) DNA quantification; (C) histological sections of fixed ECM gel drops stained with Picrosirius Red, Verhoeff's and Alcian Blue for collagen, elastin and glycosaminoglycans, respectively, scale bar 200 μm; (D) quantification of collagen, elastin and GAG; (E) analysis of the collagen types in ECM gel and Matrigel by staining for collagen I, III, and IV, scale bar 100 μm; (F) SEM images, scale bars 1 μm; (G) turbidimetry analysis during gelation; (H) storage and (I) loss modulus by oscillatory rheology; and (J) elastic modulus. Adapted with permission from Ref. [123]. Copyright 2019, Springer Nature.

Fig. 4 demonstrates an example of tissue decellularization and its morphological and physicochemical characterization [123]. The authors used a detergent-enzymatic treatment to accomplish the decellularization of porcine intestine tissue (Fig. 4A), in order to significantly reduce the DNA content (Fig. 4B) while minimizing morphological alterations (Fig. 4F). In comparison with Matrigel, a benchmark extracted matrix, the data revealed that a similar signal is obtained concerning the amount of collagen I, III and IV (Fig. 4C–E). Regarding mechanical properties, the authors observed that the ECM at a concentration of 6 mg/mL exhibited a similar rheological profile compared with Matrigel (Fig. 4G–J).

### 3.2 From cell culture

The ECM isolated from human- or animal-derived decellularized tissues is one of the most successful biomaterials in clinics. However, it presents some limitations such as its limited availability. Also, the use of xenogeneic-derived ECM as an alternative source carries increased risk of immunological incompatibility and disease transmission. Cell culture-derived ECM (CC-ECM) has the ability to overcome many of the limitations imposed by the tissue-derived ECM and possesses characteristics that enables it to recapitulate to a great extent the complex biologic system of the native tissue. As such, CC-ECM scaffolds have yielded significant results in TERM applications [134]. Briefly, *in vitro* cultures allow the continuous production of CC-ECM due to the cells’ expansion ability, while maintaining it in pathogen-free conditions. Also, ECM can be obtained from autologous cultured cells, which mitigates concerns regarding immune responses. Additionally, the secreted ECM can have directional differentiation guidance for cells, since different cell source environments have specific differentiation patterns. Furthermore, it is possible to control the cell culture conditions to achieve some desired output in terms of properties of the isolated CC-ECM. This can be performed to facilitate ECM deposition by exposing cells to precise stimuli such as supplementation with specific factors, exposure to macromolecular crowding (MMC), and by adjusting certain culture conditions. Media supplementation with factors such as l-ascorbic acid facilitates ECM deposition, since ascorbate is a cofactor of lysyl and prolyl hydroxylase enzymes, which are essential in collagen synthesis [135]. This supplementation facilitates ECM deposition, since type I collagen is the most abundant ECM component and is directly influenced by this factor, increasing the overall production of CC-ECM [136,137]. Another method to increase ECM production is by exposing the cells to MMC, which consists in introducing macromolecules (e.g., Ficoll, carrageenan, hyaluronic acid, and polyvinylpyrrolidone) in the cell culture milieu [138]. This MMC effect directs ECM production and organization, accelerating the production of ECM-rich supramolecular assemblies by imitating the dense extracellular space [139]. Furthermore, it has been shown that low serum concentrated media favors ECM deposition (<1 % v/v) when compared to high serum concentrated media, even when combined with the MMC method [139,140]. This may be due to the fact that serum has exogenous MMPs in its composition, which degrade the produced ECM and influence the natural ECM remodeling cycle. Modulation of oxygen tension, such as hypoxia conditioning, also contributes to increased release of major ECM proteins and angiogenic factors as shown in dermal fibroblasts and in mesenchymal stem cells [141]. Finally, mechanical preconditioning has also been shown to favor certain ECM proteins production in adipose-derived stem cells, and in vascular smooth muscle cells when exposed to cyclic stretching [142]. These findings have important implications when using cells that can respond to their mechanical environment in a similar way as the native ones, which varies between different tissues and organs.

While tissue-/organ–derived ECM decellularization protocols have been widely reviewed and their impact on the ECM evaluated, there are few reports overviewing the extraction impact on CC-ECM. The extraction of CC-ECM is usually performed with similar but gentler decellularization methods as for ECM derived from whole tissues-/organ. While more studies are necessary regarding the decellularization methods specific for CC-ECM and their protocol optimization, we anticipate their standardization to be a far easier matter than in the case of tissues and organs due to the relative simplicity of cell cultures. In one of the few studies on this matter, Lu and colleagues have tested seven different decellularization protocols to prepare scaffolds derived from the ECM of mesenchymal stem cells and only two methods were able to successfully remove cellular components, while preserving the ECM structure and components, as well as eliciting mild *in vivo* host responses [143]. Notwithstanding the lack of standardization in the extraction methods, CC-ECM has been explored in several contexts for TERM. These include studies regarding improvement of cellular functions, as well as recreation of cellular niches for the study of physiological (tissue and stem cell niches) and pathological (disease models engineering) contexts. Also, applications in tissue repair and regeneration were developed in numerous settings such as skeletal, cardiovascular, bone, cartilage, periodontal, skin, vascular, and peripheral nerve tissue engineering [[144], [145], [146], [147], [148], [149], [150]]. Despite these reports, relatively small advances in the use of CC-ECM have been made in the last few years, mostly due to scalability issues. ECM produced in cell cultures is relatively scarce. In our view, efforts must be made to solve this issue since the reward can be significant. Contrariwise to organ and tissue-derived ECM, less ethical issues and safety concerns are associated with CC-ECM, which is very important for the economic viability of future clinical products. Automated cell culture systems that could support ECM-producing cells in a high-throughput manner can be a way to address the issue of scalability [151].

## 4 ECM characterization methods

As already mentioned, the decellularization process of the material of interest (tissue/organ, or cell culture) unavoidably inflicts changes to the extracted ECM. For that reason, there is a need to evaluate the impact of the processing steps on the compositional and biological properties of the final product. To do so, there are several methodological analyses usually performed. Chemical and Biochemical composition, as well as the structural characterization of the extracted ECM can be evaluated by colorimetric assays, SDS-PAGE, Western Blot, immunohistochemical, proteomic, FTIR and Raman spectroscopy, and circular dichroism. The ECM physical properties can also be performed by rheology, while its thermostability evaluated by differential scanning calorimetry. Additionally, ultrastructural evaluation (morphology) of the resulting material can be performed by several microscopy techniques such as scanning and transmission electron microscopy and atomic force microscopy. Finally, functional biologic characterization of the ECM-derived construct is implemented in *in vitro* and *in vivo* experiments. These techniques are summarized, together with their main advantagess and drawbacks, in Table 3.

**Table 2:** Most common colorimetric assays available for the quantification of the major ECM components, with some of their respective specifications.

Component	Dye	Detection (Absorbance)	Action mechanism	References
Collagen	Picrosirius Red	570 nm	Hydroxyproline detection	[155]
	4-(Dimethylamino) benzaldehyde (DMAB)	530–560 nm		[156]
Elastin	5,10,15,20-tetraphenyl-21*H*,23*H*-porphine tetra-sulfonate (TPPS)	513 nm	Basic and non-polar amino acids	[157]
sGAG	1,9-dimethylmethylene blue (DMMB)	525 nm	Sulfated groups	[158,159]
	Alcian Blue	600–620 nm		[160]

**Table 3:** Summary of techniques available for ECM characterization, their specific application, and summary of advantages and disadvantages.

Technique	Quantitative	Application	Advantages	Disadvantages
**Colorimetric assays**	✓	Quantification of collagen, elastin and sGAG	- Fast; -Economic.	-Matrix-interference; -Dye inefficient stoichiometric binding; -Lack of specificity.
**Histology**	✓	Identification and localization assessment of ECM components	-Compatible with other techniques; -Cellular and structural details.	-Highly laborious protocols. -Sample fixation artifacts.
**SDS-PAGE**	✓^a^	ECM protein profile assessment	-Easy and fast; -Sample versatility and compatibility.	-Limited resolution for large proteins; -Protein overlapping.
**Western Blot**	✓	Identification of specific ECM components	-Protein specificity and sensitivity; -Quantification and detection of post-translational modifications.	-Expensive (antibodies): -Time-consuming; -Technical variability.
**FTIR/Raman microscopy**	✓^a^	Mapping-distribution of ECM-components	-Non-invasive; -Non-degrading; -Label- and artifact-free.	-Expensive (equipment); -Laborious data analysis.
**Mass spectrometry**	✓	Protein identification and quantification	-Reliable; -Highly sensitive.	-Time-consuming; -Expensive (equipment)
**Circular Dichroism**	✓^a^	Protein Secondary structure evaluation	-Easy and fast; -Economic; -Properties evaluation with different temperatures	-Limited structural information; -Complex data interpretations; -Instrumental limitations (limited buffers and sample concentration).
**Differential Scanning Calorimetry**	×	Determination of thermal properties	-Thermal transitions and stability; -Sample compatibility (solids, gels, liquids, etc)	-Complex data interpretation; -Overlapping transitions;
**Rheology**	✓^a^	Measurement of stress, strain, or viscosity	-Mechanical properties of the sample; -Non-destructive and non-invasive;	-Complex data interpretation; -Instrumentation limitations; -Time-consuming.
**Scanning Electron Microscopy**	×	Surface Morphology Porosity	-High magnification; -Cryo-SEM can be used for hydrated samples	-Samples dehydration can alter their structure; -Imaging restricted to the surface of the sample.
**Transmission Electron Microscopy**	×	Surface Morphology	-High magnification and special resolution; -Imaging of thin specimens.	-Samples processing limitations; -Time-consuming; -Expensive.
**Atomic Force Microscopy**	×	Surface Adhesion, Topology, and Morphology	-Sample versatility; -High-resolution imaging; -3D imaging of the samples' surface.	-Time-consuming; -Image artifacts; -Expensive (equipment and maintenance).
***In vitro* testing**	✓^a^	Cytocompatibility and degradation profile	-Cost-effective; -Rapid and more ethical screenings (comparing to *in vivo*).	-Limited extrapolation to living organisms; -Limited clinical relevance.
***In vivo* testing**	✓^a^	Assessment of efficacy and functionality	-Physiological relevance; -Evaluation of safety, toxicity and efficacy.	-Ethical issues; -Animal sacrifice; -Regulatory requirements; -Expensive and time-consuming.

a Semi-quantitative analysis.

**Fig. 5:**
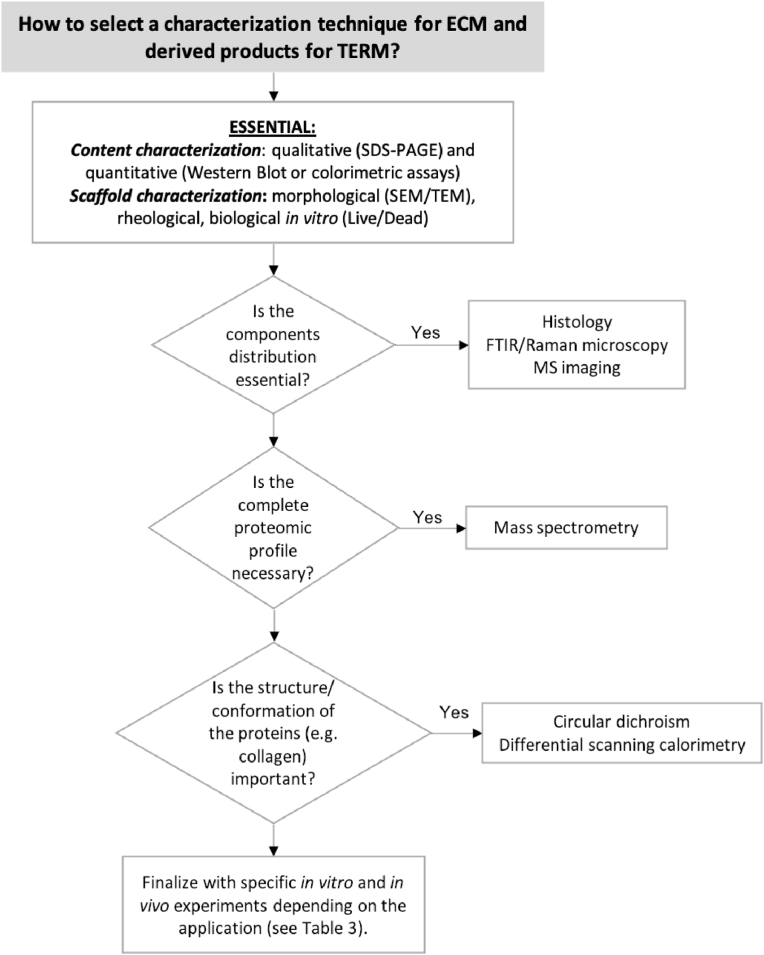
Flowchart guide to aid on the selection of the most appropriate ECM characterization techniques.

Fig. 5 provides a flowchart to aid researchers working with decellularized ECM on the selection of the most adequate characterization techniques depending to assess different features.

### 4.1 Chemical, biochemical, and structural characterization

#### 4.1.1 SDS-PAGE and western blot

To evaluate the composition after the decellularization process, SDS-PAGE (Sodium Dodecyl Sulfate Polyacrylamide Gel Electrophoresis) is commonly used for comparison of the protein profile within the ECM extract. SDS-PAGE is a technique used to separate proteins based on their molecular weight by using an electric current within a porous gel. This allows the separation of all proteins within a protein mixture such as the ECM. In fact, some studies have been performed in order to compare differences in the ECM protein profile when using the same extraction methods for different ECM origins, or to compare different extraction methods for the same ECM source [152,153]. This technique also helps in the decision of the best extraction method for the ECM in study. To verify the presence of specific proteins of interest and, if wanted, to quantify and to compare it between samples, Western blot technique can be used [154]. After running an SDS-PAGE, the proteins can be electrophoretically transferred onto a nitrocellulose or polyvinylidene difluoride membrane, and using specific primary and secondary antibodies, detect the proteins of interest by a chemiluminescent or chromogenic visualization method. When implementing this technique, researchers need to check for possible artifacts, and the analysis must be performed under very controlled laboratory conditions. In our opinion, SDS-PAGE is an indispensable technique for the characterization of extracted ECM, allowing the confirmation of the most abundant proteins with a fast and economic protocol.

#### 4.1.2 Colorimetric assays

Quantification methods based on dye-labeling techniques are one of the most explored approaches for the determination of ECM components. The quantitative analysis of the major elements, such as collagen (total, insoluble or soluble), elastin and GAGs can be obtained, with fast, reliable, economic and sensitive procedures [155,156]. Table 2 summarizes the most common approaches used, compiling some specifications associated. Currently, many protocols and kits are designed to ensure minimum interference by the other matrix components, nonetheless, this is still the main disadvantage associated with these methods.

**Fig. 6:**
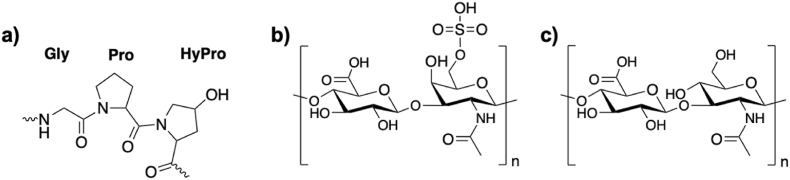
a) Major amino acid components of collagen type I represented by glycine (Gly), proline (Pro) and hydroxyproline (HyPro) [164]; **b)** chemical structure of chondroitin sulfate; **c)** chemical structure of hyaluronic acid [165].

Each dye has a distinct mechanism of action, related to site-specific reactions/binding. Collagen quantification is based on the detection of hydroxyproline (Fig. 6a), a non-proteinogenic amino acid formed by posttranslational action of the enzyme prolylhydroxylase on specific proline residues [161]. Elastin quantification uses TPPS dye, a water-soluble synthetic porphyrin, that binds to the basic and non-polar amino acids. This assay includes a hydrolysis step with oxalic acid, enabling the quantification of tropoelastins, atherogenic elastins, and α- and κ-elastins obtained from insoluble elastin [157,162]. Glycosaminoglycans, such as chondroitin sulfate, dermatan sulfate, keratan sulfate and heparan sulfate are quantified with a cationic dye through an ionic interaction with the negatively charged sulfate groups (Fig. 6b). Hyaluronic acid is the only exception, due to the absence of this anionic group (Fig. 6c) [160,163].

Colorimetric assays provide valuable information with fast procedures. Their limitations regarding matrix interference, low specificity and limits of detections can be overcome by the use of complementing techniques, detailed below. Nonetheless, colorimetric assays provide, from our point of view, an excellent complement the data obtained from SDS-Page and Western Blot.

#### 4.1.3 Histology

**Fig. 7:**
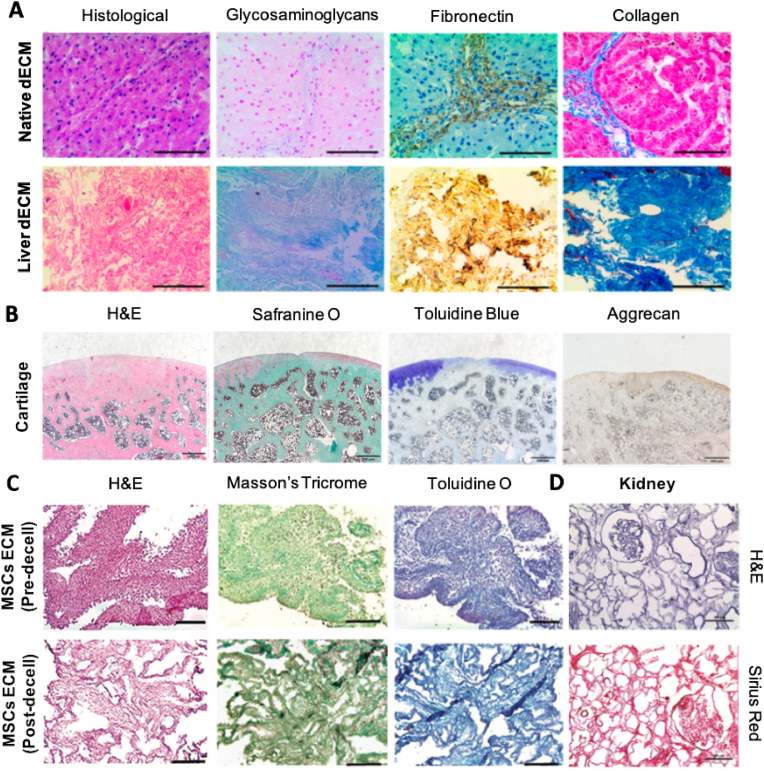
Histological staining of different ECM components from different sources: (A) Comparison between native and decellularized ECM liver, to observe the histological morphology (H&E), and distribution of GAGs (alcian blue), fibronectin (immunohistochemistry) and collagen (Masson's trichrome). Adapted with permission from Ref. [169]. Copyright 2017, American Chemical Society. (B) Cartilage tissue construct, depicting the histological morphology (H&E), collagen in green (Safranine O), proteoglycans (Toluidine Blue), and a specific proteoglycan, Aggrecan (immunohistochemistry). Adapted with permission from Ref. [170]. Copyright 2022, Springer Nature. (C) Comparison between pre-decellularization and post-decellularization of an MSC-derived ECM, to observe the histological morphology (H&E), collagen (Masson's Tricrome), and GAGs (Toluidine O). Adapted with permission from Ref. [171]. Copyright 2021, John Wiley & Sons. (D) Decellularized kidney tissue illustrating the histological morphology (H&E) and collagen (Sirius Red). Adapted with permission from Ref. [167]. Copyright 2015, Verduci International.

ECM histological analysis is a qualitative way to assess ECM individual proteins and to determine their presence and localization. To analyze tissue samples through histology, samples normally need to be embedded in resin, OCT or paraffin wax, and cut into thin slices prior to staining. Stainings such as Haematoxylin and Eosin (H&E) are commonly used to assess decellularization efficiency, since haematoxylin stains nuclei in a blue color, while eosin stains the cytoplasm and ECM with a pink color. This allows the detection of nuclei after decellularization protocols [166]. Special stainings can be used to further characterize the ECM, allowing the detection of specific ECM components. For the selective detection of collagen networks, PicroSirius Red, also known as Sirius Red (Fig. 7D), is commonly used [167]. On the other hand, Alcian Blue allows the detection of GAGs and acid mucins. Other staining strategies can be observed in Fig. 7.

For a more fine and specific localization of proteins, techniques such as immunohistochemistry (tissue level) or immunocytochemistry (cell level) are used. This technique involves the use of antibodies, that form a complex with specific antigens, allowing the detection and visualization of the ECM components with more resolution [168]. Histological analysis have helped, for instance, in understanding the preservation of ECM components in hydrogels when using different decellularization protocols [116].

In Fig. 7, different examples of histological staining of tissues (liver, kidney, and cartilage) and mesenchymal stem cell (MSC)-derived ECM can be observed. As previously mentioned, and depicted in Fig. 7, H&E is the standard staining method of choice to assess the success of the decellularization.

Lee et al. resorted to immunohistological staining of liver tissue to evaluate the effect of the decellularization process on the main ECM components. The authors used alcian blue, anti-fibronectin antibody, and Masson's trichrome staining to observe GAGs, fibronectin, and collagen, respectively (Fig. 7A). Comparing native vis-à-vis decellularized ECM, all components were well preserved [169]. After production and implantation of an ECM-derived hydrogel, Zeng and coworkers used H&E and Safranine O staining of rat knee joint tissue sections to observe the distribution of chondrocytes and collagen, respectively. Toluidine blue staining was used to observe the overall proteoglycans in cartilage, while immunochemistry for Aggrecan, the most abundant proteoglycan in cartilage, was applied in the defect area (Fig. 7B) [170]. Antich and collaborators used MSC-derived ECM for cartilage tissue engineering. The authors resorted to histology to observe the impact of the decellularization method on the ECM, using H&E (cellular content), Masson's Tricrome (collagen) and toluidine O (GAGs) (Fig. 7C) [171].

#### 4.1.4 FTIR and Raman spectroscopy

**Fig. 8:**
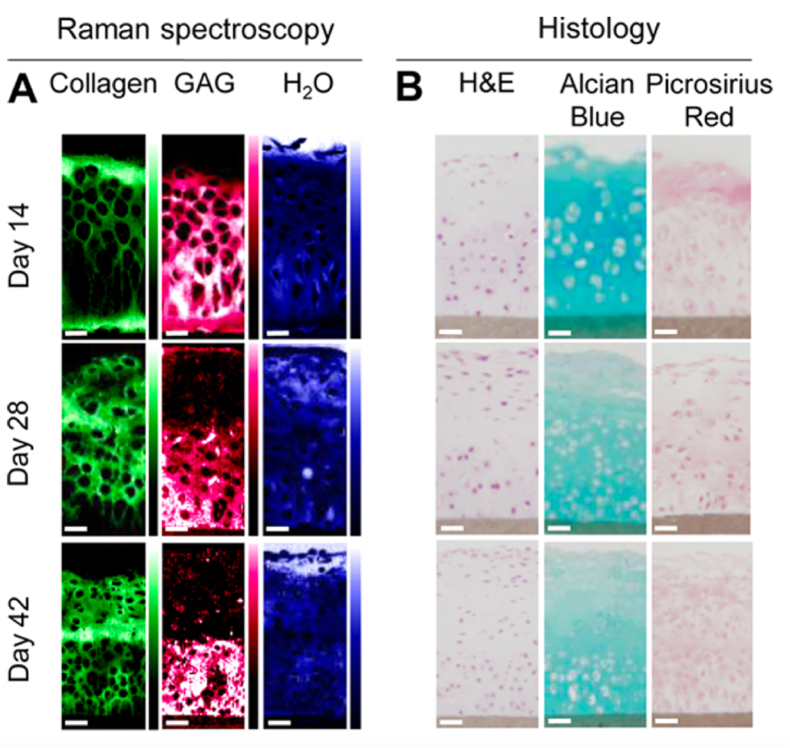
Comparison of biochemical distributions in tissue-engineered constructs measured by Raman spectroscopy and Histological analysis. (**A**) Distribution images of ECM components collagen, GAG, and H_2_O, obtained using Raman spectroscopic imaging and MCR analysis for each incubation time (i.e., 14, 28, and 42 days). (**B**) Representative H&E, Alcian blue, and Picrosirius red stained histological slides for each incubation time. Adapted with permission from Ref. [179]. Copyright 2016, American Chemical Society.

Fourier-transform infrared spectroscopy (FTIR) and Raman spectroscopy are the most important vibrational spectroscopic techniques used for chemical and structural elucidation. They complement each other since transitions allowed in FTIR are forbidden in Raman, and vice-versa [172]. FTIR measures the vibrational absorption of compounds [173], focusing on the asymmetric vibrations of polar groups [172]. Raman spectroscopy relies on the inelastic light scattering produced through the interaction between light and molecules (Raman effect), allowing the identification of nonpolar groups [172]. The combination of a microscope with a vibrational spectroscope (FTIR or Raman imaging) offers valuable insights regarding composition within microscopic points. The multiple microscopic objectives available enables the acquisition of maps, providing information on the spatial distributions of ECM components across a sample. Chrabaszcz et al., used FTIR spectroscopy imaging (FTIR-I) to observe changes in ECM proteins of the lungs. The authors observed not only the different types of proteins but also their degree of degradation due to breast cancer metastasis in a mice model [174]. In a different work, FTIR-I was used for the characterization of cartilage-to-bone transition in terms of collagen, proteoglycan and mineral distribution [175]. A similar strategy was implemented by Spalazzi et al., across the ligament-to-bone insertion [176]. Apart from providing valuable information, FTIR-I exhibits several limitations compared to Raman microscopy (RM), such as relatively low resolution and incompatibility with ECM aqueous samples [173]. RM has drawn a lot of attention in recent years in the field of ECM characterization. It offers unique insights into the composition and structure at a molecular level of tissues and cells with an outstanding degree of biomolecular specificity. Furthermore, RM can mitigate several limitations associated with histological and immunohistochemical evaluations [173]. Microstructural fluctuations regarding orientation, inclusions, composition, or stress are also accessible by RM [172]. Albro and coworkers used this technique in combination with a multivariate curve resolution (MCR) for the quantitative analysis of ECM constituents (GAG, collagen, and water) of native and engineered cartilage tissue [177]. Littmann et al. employed RM for the semi-quantitative analysis of GAG, collagen, and lipid content of human mesenchymal stem cell ECM [178]. Bergholt and coworkers used RM for the elucidation of articular cartilage and tissue-engineered constructs components-organization (Fig. 8), highlighting the potential of RM for the analysis of complex samples [179].

The use of this technique for characterization and diagnostics purposes through ECM analysis has grown exponentially in recent years, revealing itself by its use in *in vitro*, *ex vivo* and *in vivo* samples [173]. Furthermore, since RM relies on the individual component spectrum, and histology in dyes, we may conclude that RM offers a greater degree of flexibility. As a single equipment, it has the capability to provide the distribution analysis of multiple proteins and other molecules, which is not easily achievable through histology alone.

#### 4.1.5 Mass spectrometry

Mass spectrometry is by far the most complete technique regarding ECM characterization. It allows the identification and quantification of all proteins in a sample with a remarkable degree of reproducibility and sensitivity. The wide range of ECM applications has driven a massive number of reports on this subject [180]. A detailed proteomic analysis of ECM can reveal relevant and tumorigenesis-promoting proteins, playing a crucial role in therapeutics and preventive medicine [181]. As previously stated, ECM proteins (collagens, glycoproteins, proteoglycans, enzymes, etc.) represents the main ECM constituents, leading to a matrisome composed of over 100 proteins [182]. Envisioning a deeper and more complete understanding of ECM functionality, several reports on proteomic protocols for that purpose have been recently published [[183], [184], [185], [186], [187], [188]].

**Fig. 9:**
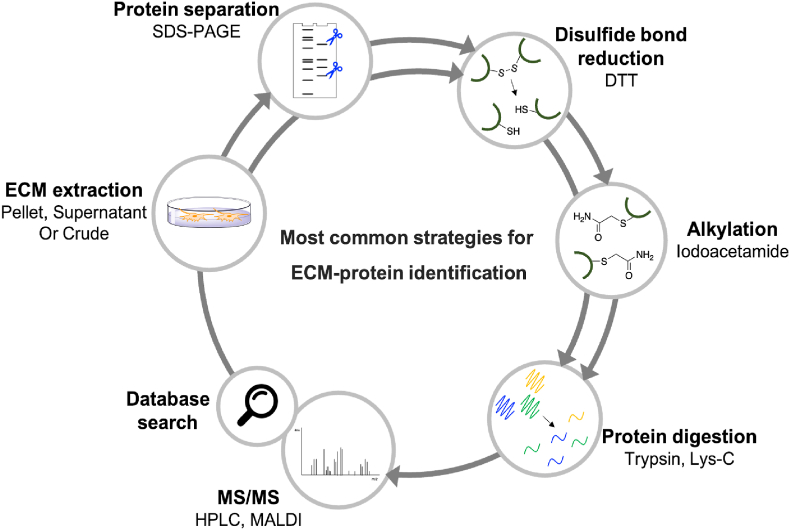
Most common strategies reported for the identification of ECM proteins.

The extraction of the ECM, using previously described strategies, is the first step, and different parts can be submitted for analysis (pellet, supernatant, or crude). An accurate decellularization step is essential for the detection and identification of lower-abundance proteins [183]. Thereafter, disulfide bond reduction is performed, preceded or not, by protein separation (ex: 1D or 2D Electrophoresis). Cysteine alkylation is usually performed immediately to prevent further oxidation. The final step evolves enzymatic (trypsin, Lys-C, etc.) or chemical (CnBr) digestion into peptides. An additional step with PNGase F can be followed, for the hydrolysis of *N*-linked oligosaccharides from glycoproteins. The samples are then analyzed by mass spectrometry followed by a database search (Fig. 9). Each protein is digested into unique peptide sequences, enabling an accurate identification. A precise description of the posttranslational modifications is crucial within this step [188]. Other components of ECM, such as the disaccharide content, from glycosaminoglycans, can be identified by LC-MS/MS [189].

##### 4.1.5.1 Mass spectrometry imaging (MSI)

Mass spectrometry provides detailed proteomic information. However, the link between the -omics and morphology is lost during sample preparation. MSI has emerged to mitigate this issue, affording valuable information regarding the quantity and distribution of peptides, proteins, lipids and metabolites across a biological sample [190]. Previously, we described the application of vibrational spectroscopic techniques coupled to a microscope for a similar purpose. However, MS tools are more sensitive, enabling the detection of specific proteins within a spatial region. Apart from the significant investment and advances in this field, to date, there is no enzymatic technology that allows the elucidation of all ECM constituents at once [191]. Angel and coworkers, used matrix-assisted laser desorption/ionization imaging mass spectrometry (MALDI-IMS) for the spatial localization of elastin and collagens sequences on formalin-fixed paraffin embedded tissues [192]. The authors digested the proteins with enzymes (elastin and collagenases), enabling their spatial detection. The findings unraveled the use of MALDI-IMS for diagnostics, prognostics and therapeutics. In a recent work, chondroitin sulfate, *N*-glycans and other GAGs and peptides were successfully quantified and detected across a tissue section with MALDI-IMS, by the application of chondroitinase ABC, elastase, PNGase F and collagenase type III as the enzymatic cocktail [191]. To address the limitations regarding sensitivity, Piehowski and coworkers, developed a microfluidic platform that extensively increased the proteomic coverage of small samples. The authors were able to quantify and observe the distribution of >2000 proteins in a 100 μm tissue section [193]. While these findings demonstrate the potential of MSI for ECM characterization, continuous efforts to improve the sensitivity of this technology must be employed.

#### 4.1.6 Circular dichroism

Circular Dichroism (CD) is a light absorption spectroscopy method that allows the study of proteins' secondary structure, their interactions with other molecules, folding, stability, and ligand binding properties [194]. This method is based on the difference of (left- and right-handed) circularly polarized light absorption by optically active molecules to determine their molecular configurations [195]. It also allows to monitor the effect of an environmental change (e.g., pH, temperature) on the secondary structure of the molecule of interest without having to resort to more expensive techniques. In the case of ECM proteins, CD is able to characterize and quantify the secondary structure content in the far-UV wavelength (260 - 180 nm). It is possible to classify the structural protein content in terms of α-helix, β-sheet, and random coil [196]. The CD can also detect the aromatic residues (e.g., tryptophan, tyrosine, and phenylalanine) signal in the near-UV wavelength (260–300 nm), which can give information regarding the protein tertiary structure. Furthermore, due to the possibility to perform temperature ramps, gelation studies can be accomplished, where the effect of temperature on the secondary structure of the ECM proteins can be perceived [197].

In the ECM context, as previously mentioned, collagen is the most predominant protein in its composition, and it presents a distinctive triple helix conformation. This structure exhibits distinct CD transitions, typically presenting a positive peak at 222 nm, and a negative peak around 197 nm, which is characteristic of the triple helical structural conformation of collagen [196]. In fact, Drzewiecki and colleagues have demonstrated that this technique can be used to evaluate proteins secondary structure as well as to monitor collagen fibrillogenesis [198]. This technique was also employed to assess the impact of the treatment with HHP for corneal decellularization on the collagen triple helical structure by Hashimoto and coworkers [199]. One limitation associated to CD is the limit of the High Tension voltage, which differ depending of the instruments. This restricts the type of buffers that can be used, and the range of protein concentrations, due to their high ability to absorb light [197].

### 4.2 Physical characterization

#### 4.2.1 Differential scanning calorimetry

Differential Scanning Calorimetry (DSC) is a thermoanalytical method used to measure the stability of a biomolecule such as proteins in its native form. DSC measures the heat exchange associated with transitions induced by temperature change, which are correlated with the protein thermal denaturation or “thermal melting”. In the case of proteins, DSC can be used as an indicator for ligand binding, to assess thermodynamic stability and its folding mechanism [200]. Basic DSC measurements are performed by inducing constant temperature increase both in an empty reference cell or that contains the solvent, and a sample cell that includes the sample of interest (anhydrous or in the same buffer), and monitoring the temperature difference between the cells. The temperature increase induces processes in the sample cell, that requires more energy to bring the sample to the same temperature as the reference cell. This results in a thermal gradient that is converted into power, which controls the device in order to return the temperature difference between the cells to 0 °C. DSC devices measure this heat uptake from the sample cell, which gives information regarding protein stability [200]. The higher the thermal transition temperature of a protein, the more stable it is. In fact, Sun and Leung used this technique to assess human dermis ECM degradation and instability after gamma irradiation [201]. Liu and collaborators used DSC as a complementary technique to evaluate the optimum conditions for the preparation of acellular dermal matrix [202]. DSC also allows the determination of the thermal transition temperatures of samples in solutions, solids, and in suspensions.

#### 4.2.2 Rheology

Rheology is the study of the flow and deformation of materials under applied stresses or forces. It focuses on understanding how materials respond to different types of mechanical forces and how their physical properties affect their flow behavior [203]. One of the most important properties that rheology assesses is the viscoelastic character. Elasticity is the ability of the material to recover its original shape after deformation, while viscosity is the ability to flow and dissipate energy. The ECM exhibits both elastic and viscous characteristics, attributed to the presence of proteins like collagen and elastin, which can undergo deformation and relaxation. Another important rheological property of ECM that needs to be assessed is stiffness or rigidity, which affects the cellular behavior and tissue function. Stiffness is determined by the composition of the ECM and the organization of its components, e.g. collagen imparts a stiffer behavior, while a higher proportion of proteoglycans can impart greater compressibility and flexibility [204]. Furthermore, to assess if the ECM acquires gel-like properties at 37 °C, its storage modulus (G′) needs to be higher than the loss modulus (G″) [123].

The rheological properties of ECM influence cell behavior, including, migration, differentiation, adhesion and proliferation. Cells can sense and respond to the mechanical cues provided by the ECM, modulating their functions accordingly. Therefore, understanding the rheological properties of ECM and ECM-derived scaffolds is crucial and plays an important role in the TE process since different native tissues have different rheological properties and, thus, different TERM applications will require different rheological properties [205]. These can be measured using different instruments and techniques such as a rotational or oscillatory rheometer or by dynamic mechanical analysis, measuring their response to stress, strain, or viscosity [206].

Antich and coworkers used rheological measurements to evaluate the injectability and gelling abilities of mesenchymal stem cells derived ECM. Their data revealed that the hydrogels produced revealed a thixotropic behavior [171].

Giobbe et al. compared the rheological and mechanical profiles of decellularized ECM from porcine small intestine mucosa/submucosa with Matrigel. Their findings show that a 6 mg/mL of dECM, a similar rheological profile in terms of storage and loss modulus to that of Matrigel was obtained [123].

### 4.3 Morphological characterization

The ultrastructure of the ECM and of ECM-derived scaffolds is also a determinant of their performance within living systems. Therefore, high-resolution information on the ECM ultrastructure and its interaction with the surrounding microenvironment is very important. As such, several imaging techniques have been used to study the morphological and topological characteristics of the ECM and of ECM-derived scaffolds, which includes scanning and transmission electron microscopy (SEM and TEM), and atomic force microscopy (AFM).

#### 4.3.1 SEM

**Fig. 10:**
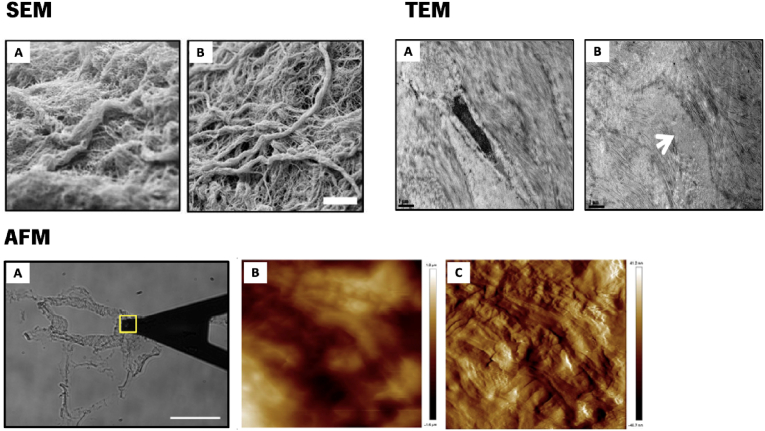
SEM images of (A) fresh human Wharton's jelly, and (B) decellularized Wharton's jelly matrix, scale bar 5 μm. Adapted with permission from Ref. [209]. Copyright 2020, Frontiers. **TEM images** of (A) fresh human lenticules, and (B) decellularized lenticules. Scale bars: 1 μm. Adapted with permission from Ref. [214]. Copyright 2022, Elsevier. **AFM images** of decellularized mouse lung, (A) Bright-field optical image of a decellularized lung section showing the wall of a vessel probed with AFM; (B) Topography and (C) deflection AFM images, scale bar = 50 μm. Adapted with permission from Ref. [215]. Copyright 2017, John Wiley and Sons.

SEM is a powerful tool that can create 3D images by scanning the surface of a sample through a focused beam of electrons. It provides a high-resolution image within a nanometer scale, where its magnification can reach up to 2 × 10^5^ times [207]. Detailed information regarding the surface topography, morphology and composition of the sample can be achieved. Sample preparation typically consists on an array of protocols with the aim of samples' structure stabilization, dehydration and coating to ensure samples adequate conductivity. Samples dehydration is required since the water content of the sample can interfere with the vacuum inside the equipment and influence the final analysis. This dehydration requirement can alter the samples morphology and structure. However, it is possible to use cryo-SEM to study hydrated samples [116,208]. This method allows to maintain the water content inside the sample. Essentially, the samples are frozen under high pressure, avoiding evaporation of the water molecules. However, ice crystal formation is a setback from this technique. Penolazzi and colleagues used SEM to verify the maintenance of the ECM fibrillar structure after the decellularization of human Wharton's Jelly matrix (Fig. 10) [209].

#### 4.3.2 TEM

Although SEM is the most common electron microscopy technique used, it can only be used to visualize the surface of the sample in question. TEM, on the other hand, is a technique that allows the detailed visualization of structures in the interior of the sample, at an even higher resolution than SEM. This is done through an electron beam that is transmitted through a thin sample slice. With these properties in mind, TEM can be used to investigate, for e.g., the interaction between nanoparticles with subcellular structures. The interaction between cells and the ECM can be visualized by TEM, allowing a more advanced visualization of the ECM patterning, even on a specific protein (e.g., collagen) arrangements level [210,211]. However, this technique is not used as often as SEM or AFM, since it requires the complex processing into thin slices (<100 nm) of the biological samples. This can limit the visualization of the samples structure and the sample processing is time consuming and it doesn't allow dynamic studies due to samples fixation and resin embedding.

#### 4.3.3 AFM

AFM is a technique used for the characterization of the mechanical properties of soft matter such as ECM or ECM-derived scaffolds. It allows the assessment of Young's elastic modulus (E), which defines the samples resistance to distortion. AFM can assess E and other mechanical properties at a nanometer and micrometer scale, which is relevant for the mechanical interactions that occur between the ECM and cells. This technique is based on the use of a nanometer/micrometer sized tip at the end of a flexible cantilever that acts as a force sensor [212]. The movement and distortion of the cantilever results from the local tip-sample force interactions, where the computation of the local sample deformation allows to assess the local value of E, allowing to trace a surface profile of the sample. In fact, Zhou et al. mapped the surface profile and elastic modulus from bovine cortical bone and its surrounding matrix, showing the collagen fibril bundles, different degrees of mineralization and fibril orientations [213]. One important advantage of this technique is the possibility to examine samples in liquid within physiologically relevant temperatures, making AFM suitable for live biological applications.

### 4.4 Biological characterization

#### 4.4.1 *In vitro* and *in vivo* testing

The ultimate characterization of ECM and ECM-derived scaffolds is to assess its functionality, biocompatibility and regenerative potential when interacting with biological systems. To do so, *in vitro* and/or *in vivo* testing needs to be performed.

Assessing the biodegradability behavior of ECM-derived materials upon contact with physiological media can provide predictive information regarding their lifetime in a biological environment. Assays to test this may be performed using enzymes, such as collagen or lysozyme, or only enzyme-free physiological media, and changes in the materials’ mass or structural integrity over time are monitored [216].

*In vitro* assays can also provide important information regarding the viability, morphology, proliferation and differentiation of cells in different substrates, which can give substantial cues regarding the functionality of ECM and ECM-derived scaffolds [217,218]. Cultured cells can be seeded onto the ECM-derived materials, and techniques such as MTT or MTS assay, Live/Dead staining or cell counting can assess the basic cellular response in terms of, respectively, metabolic activity, viability and proliferation during the interaction with the ECM materials [219]. The choice of cells to be seeded into the ECM scaffold depends on the envisaged application. Most researchers use stem cells, such as human mesenchymal stem cells (hMSCs) or human induced pluripotent stem cells (hiPSCs), due to their ability to differentiate into other cell types. hMSCs are already used in many biomedical applications owing to their safety and hypoimmunogenicity, while hiPSCs allow the possibility for autologous transplantation [220].

While *in vitro* testing provides valuable preliminary data, it cannot fully mimic the complex environment in a living organism. *In vivo* testing is a necessary step that provides a more comprehensive understanding of the interaction between ECM-derived materials and the tissues of a living host. Such assays provide vital information such as the *in vivo* biocompatibility of the ECM-derived materials in terms of the host immune response, target tissue regeneration and overall integration within the host tissues. *In vivo* assays in this context typically entail the implantation studies in animal models. First, an accurate choice of the animal model must be carefully deliberated, considering the specific research objectives and the targeted tissue or organ, and strictly following ethical considerations and regulatory guidelines. After implantation, histological analysis of sections of tissues excised from the area near the implanted biomaterial is performed. This analysis provides an overall picture of the host immune response the ECM-derived biomaterial so as to determine its biocompatibility and integration with the host's tissue. The assessment of its regeneration potential and functionality is further performed through the observation of new tissue formation, angiogenesis and morphology by resorting to other already described techniques such as immunohistochemistry [[221], [222], [223]].

It is important to state that the *in vitro* and *in vivo* experiments to be conducted are highly dependent on the type of TERM application. In our opinion, *in vitro* assays such as biodegradability, and the evaluation of cells’ viability, metabolic activity, and proliferation are crucial and must be performed independently of the final application.

If further characterizations are pondered/needed, specific experiments are available, depending on the application. For instance, calcium mineralization, assessed by staining with Alizarin Red S is usually performed for bone applications. Immunocytochemistry (ICC) or immunohistochemistry (IHC) for specific osteogenic markers such as osteopontin and osteocalcin can be made. Commonly, qPCR is also performed, to measure the gene expression levels of osteogenic genes such as alkaline phosphatase (ALP), bone morphogenetic protein-2 (BMP-2), osteocalcin (OC), and osteopontin (OPN) [224,225]. Regarding cardiac applications, IHC or ICC analysis for markers such as α-sarcomeric actin and cardiac troponin I is the most standard approach. Other analysis, such as Ca^2+^ imaging and the analysis of the vascularization capability can complement the characterization [220,226]. For adipose tissue, gene expression levels of lipoprotein lipase (LPL), peroxisome proliferator-activated receptor gamma (PPARγ) and CCAAT/enhancer binding protein alpha (C/EBPα) can be evaluated [227]. The quantification of lipidic deposits in the constructs may be performed by staining with dyes such as Oil Red O [228].

These and other characterization protocols applied for specific TERM applications are compiled in Table 4.

**Table 4:** Compilation of specific characterization techniques and assays performed for different TERM applications of ECM-derived scaffolds.

TERM application	ECM source	Scaffold produced	In vitro assays	In vivo assays	Other characterization techniques	Reference
Gastric, hepatic, pancreatic and SI	Porcine intestine tissue	Hydrogel	DNA concentration Live/Dead	CAM assay Subcutaneous grafting into NSG mice	Colorimetric assays Histology Turbidity Rheology SEM Proteomic	[123]
Cornea	Porcine cornea	Electrospun	Col I (IHC) Quantitative PCR (ALDH3A1, Col I, Smooth Muscle Actin and GAPDH) Cell migration	N/P	FTIR Mechanical testing Contact angle SEM	[229]
Bone	Porcine bone	Bioprinting	Live/Dead MTT assay Alcaline phosphatase activity Alizarin Red S Staining Osteopontin expression RT-PCR (ALP, BMP-2, OCN, OPN)	N/P	Rheology Compression test FTIR	[224]
	hTMSCs from inferior turbinate tissue	Bioprinting	DNA concentration Osteogenic differentiation capability by RT-PCR (ALP, OC, OPN) Micro-CT	Implantation into calvaria defects of Sprague Dawley® rats	Colorimetric assays SEM	[225]
Cardiac	Heart tissue of Korean domestic pig	Bioprinting	Vascularization capability (extrusion) RT-PCR VEGF quantification (ELISA) α-sarcomeric actin and cardiac troponin I (IHC)	Subcutaneous implantation into Balb/c nude mice Echocardiography (Ejection fraction and fractional shortening)	N/P	[226]
	Porcine	Electrospun	Alamar Blue (cell viability) cTn1, α-sarcomeric actin, and connexin-43 (IHC) Ca^2+^ imaging Immunogenicity studies (secreted NO) RT-PCR (TNF-α and IL-1β) Total RNA	Subcutaneous implantation into C57 black mice	SEM Tensile strength Contact angle FTIR TGA Proteomic Histology	[220]
Vascular	Banked porcine smooth muscle cells	Direct use	SMC α-actin, vimentin, CD31, CD144, and vWF (IHC) Western Blot	Implantation into carotid artery of porcine	Mechanical testing of the vessels	[230]
	Dermal fibroblasts or saphenous vein fibroblasts	Direct use	SM α-actin, calponin (IHC)	N/P	Histology Tensile strength Estimated burst pressure Contractile capability	[231]
Skin	Porcine dermis	Hydrogel	DNA concentration Live/Dead Biodegradation Angiography (Micro-CT) Multicolor Flow Cytometry	Hydrogels dressings into Sprague-Dawley rats	Histology SEM Rheology Proteomics Western Blot	[232]
	Porcine dermis	Direct use	DNA concentration VEGF, FGF, TGF-β quantification (ELISA) Live/Dead	N/P	Histology Immunofluorescent staining of proteins SEM Tensile strength Suture retention test	[233]
Cartilage	Mesenchymal stem cells	Hydrogel	Live/Dead Cell proliferation Gene expression analysis Col I and II, Aggrecan (IHC)	Subcutaneous injection into CD1 (ICR) mice	Rheology Histology Colorimetric assay Proteomics SEM	[171]
	Goat ears	Direct use	DNA concentration TGF-β and TGF-β1 (ELISA) Biodegradation MTT assay Cell differentiation Immune response (IL-2, IL-4 and INF-γ) Micro-CT	Subcutaneous insertion into New Zealand white rabbits	Histology SEM Colorimetric assays Swelling properties Indentation testing	[234]
Muscle	Porcine rectus abdominis	Direct use	Integrity test (dye infusion) Vascular corrosion casting DNA concentration Cell migration Myogenic differentiation Metabolic activity (Alamar Blue)	Sprague-Dawley rats	SEM Tensile strength Colorimetric assays Histology	[235]
	Skeletal muscle from New Zealand White Rabbits	Hydrogel	DNA concentration Live/Dead Cell proliferation and differentiation Myosin heavy chain (IHC)	Surgical defect in the TA muscle of New Zealand White Rabbits	SEM Histology Colorimetric assays Rheology	[236]
Adipose tissue	Adipose tissue	Bioprinting	Live/Dead Macrophage infiltration (M1 and M2) Angiogenesis RT-PCR (LPL, PPARγ, GAPDH, C/EBPα)	Implantation into SD rats	Compression test SEM Histology	[227]
	Human adipose tissue and porcine heart tissue	Bioprinting	RT-PCR (LPL, PPARγ, GAPDH, C/EBPα) vWF and α-SMA vessel densities (IHC) Col IV and LPL (immunofluorescence staining) Apoptosis of seeded cells Quantification of accumulative lipids (Oil Red O dye)	Subcutaneous implantation into immune-deficient nude mice	SEM Colorimetric assays Microarrays Histology	[228]

N/P: Not performed.

## 5 TERM applications of ECM-derived components

ECM-derived biomaterials for TERM applications have been progressively more explored either as isolated ECM components or integrated with other biomaterials. In general, several key considerations are important when designing a scaffold for TERM: biocompatibility, biodegradability, mechanical properties, scaffold architecture, and manufacturing technology. ECM-derived biomaterials automatically meet the first two requirements because they are cell produced. However, to engineer scaffolds with appropriate mechanical properties is one of the biggest challenges when using ECM-derived biomaterials. Usually, using crosslinking agents (e.g., glutaraldehyde, genipin, transglutaminase) or combining the ECM components with other materials addresses that challenge [216]. One of those examples, is the combination of ECM-derived proteins with ceramic composites, using the tape-casting process to produce thin films [237].

The architecture of the created scaffolds is important since it should have an appropriate pore size and structure to allow cell penetration and migration in the scaffold and allow the diffusion of nutrients and waste products in and out of it. Cells interaction with the material surface is also critical since cell processes such as migration requires ligand binding. In the case of ECM-derived products, they naturally possess these ligands in the form of RGD (Arg-Gly-Asp), or other cell adhesion motifs sequences, inherently present in ECM proteins [238,239]. Finally, the manufacturing technology should be cost effective and able to be scaled-up, with the final product possessing good manufacturing practices (GMP) standards in order to become commercially available. In our opinion, this is the major drawback associated with ECM-derived biomaterials. Problems associated with the non-standardization of their isolation and extraction, and the time spent in their characterization is an overall time-consuming and expensive process, hampering their production and commercialization in the future. Furthermore, batch-to-batch variability is also one of the major concerns. Yet, the remarkable development in ECM biomaterials over the last years shows that these problems can be easily overcome by the scientific community. The use of ECM-derived materials in TERM has progressively developed from simpler 2D models using cell culture plates coated with ECM components to more advanced 3D ECM-mimicking models. Decellularized tissues/organs can be engineered in order to recapitulate the composition, structure, dynamics, and inherent cues of the ECM. In this section, we will overview commonly used scaffolds engineered from ECM-derived biomaterials using different techniques, namely hydrogels, bioprinted scaffolds, and electrospun scaffolds.

### 5.1 Hydrogels

Among the several ECM-scaffold types studied for TERM applications, hydrogels are by far the most investigated. A hydrogel is defined as a highly hydrated 3D network that maintains structural integrity due to chemical or physical reticulation between polymer/protein chains [240]. This 3D highly hydrated structure allows hydrogels to retain significant amounts of water, biological molecules, among others. Hydrogels can be classified according to their source, ionic charge, polymerization method, physical characteristics, or cross-linkers, which affects the choice of the biomaterial for a certain application [241].

ECM-derived hydrogels have a great potential for TERM applications since ECM solutions can self-assemble into a hydrogel upon incubation at physiological temperature (37 °C) or injection *in vivo*. These hydrogels present other advantageous properties such as improved biocompatibility, biological recognition, and biodegradability without presenting toxicity. Also, similarly to native ECM, ECM-derived hydrogels are capable of presenting high flexibility and elasticity, which allows nutrient diffusion and provides the necessary support for embedded cells, facilitating the complex processes important for tissue formation, repair and regeneration [242]. ECM-derived hydrogels can be produced from the ECM of any tissue of the body or from cell culture, following the already described process of decellularization and further processing to generate, for e.g., an injectable material. The production of injectable hydrogels is advantageous since their implantation is less invasive and may conform to more irregular shapes than implanted scaffolds [243]. The processing of the ECM derived from decellularized material can be performed by two main approaches. The first approach involves the grinding of the ECM into a fine powder and its suspension in liquid for further injection [244]. The second approach is based on pepsin solubilization as described by Freytes et al. [245]. The ECM is suspended in an acid solution of pepsin and enzymatically digested. Following digestion, the pH of the solutions is neutralized to the physiological level to match *in vivo* conditions and to inactivate the pepsin enzyme. The ECM hydrogels' biochemical, topological, and viscoelastic characteristics are influenced by the tissue of origin and decellularization technique [246]. ECM hydrogels can be used as substrates to promote the culture and maturation of cell lines, primary cells, and stem or progenitor cells [118,247]. They can be utilized in 2D cultures as coatings for tissue culture plastic, and as 3D gels for 3D cell culture [248]. Regarding the latter, ECM-derived hydrogels from decellularized porcine small intestine mucosa/submucosa have been shown to provide support for cell growth, viability, and endoderm-derived human organoid expansion in culture [123]. Kim and colleagues demonstrated that using ECM hydrogels from decellularized gastrointestinal tissues showed comparable and even superior results in producing suitable 3D organoid culture for tissue regeneration, drug development, and for the modeling of gastrointestinal diseases, when compared to the current gold standard Matrigel [102]. Moreover, ECM hydrogels have been used for the study of several preclinical applications, such as traumatic brain injury [249], cardiovascular dysfunctions [250], endometrial pathologies [251], cartilage defect treatment [252], and corneal regeneration [253].

Other avenues for ECM hydrogels production and processing may be implemented. For instance, through their freeze-drying, sponges or films can be produced. Wang and collaborators used decellularized porcine skin to produce ECM-hydrogels. By changing the concentration of PBS, the authors were able to produce different ECM scaffolds, such as hemostatic sponges or anticoagulation films, showing regulable coagulant/anticoagulant properties, without resourcing to any crosslinking strategy or other biomaterials [254]. ECM sponges are also commonly used for skin repair and regeneration [122]. Seo et al. have combined collagen with ECM secreted by human skin fibroblasts to produce sponges. Their data reveals the potential of such scaffolds for wound dressing applications [255].

Importantly, to facilitate the clinical use of these scaffolds, their characterization should be thorough, suitable source tissues must be chosen for particular therapeutic applications, and processing and sterilizing techniques must be improved. Finally, the application potential of ECM-derived hydrogels is increased by their compatibility with a number of production techniques such as 3D bioprinting and electrospinning.

### 5.2 3D bioprinted scaffolds

ECM-derived hydrogels have been used in a variety of applications and configurations, as described in the previous section, and, more recently, as bioinks for 3D bioprinting. 3D bioprinting is a multipurpose technique that effectively creates tissue constructs with precise and complex structures by using computer-designed models and bioinks [256]. This technique uses a layer-by-layer precise positioning of the biological components present in the bioinks, allowing the control of multiple scaffold characteristics such as architecture, composition, pore size, and surface chemistry [257]. Depending on the working principle, 3D bioprinting is divided into several categories, although the extrusion, droplet, and laser-assisted bioprinting techniques are the most frequently described [258]. Extrusion-based 3D bioprinting, also known as direct ink writing, is based on applying pressure, either by pneumatical or by mechanical force, in order to push out the bioink through a printhead, depositing it as continuous filaments on a building substrate [259]. During the process, there is a set up controlling the overflow of bioink deposition, which is an important issue when working with highly viscous solutions. Such 3D bioprinters can have multiple nozzles and print heads, which allows the deposition of multiple bioinks and the creation of multi-tissue constructs [260]. Among its advantages are low printing costs, simplicity of use, ability to support high cellular densities, and ability to use various biomaterials as bioinks [261]. However, cell viability may be impacted when the print pressure and shear force increase. Furthermore, printing resolution is relatively low. Droplet-based 3D bioprinting, also known as inkjet bioprinting, works by the release of bioink droplets from the printhead through the generation of thermal or piezoelectric forces [262]. The demand for bioprinters based on this technique has grown as a result of their reasonable cost, compatibility with living materials, and rapid bioink droplet deposition with good printing resolution [260]. The structures that can be bioprinted and the bioink's cell density are constrained by the requirement for low viscosity bioinks. The unpredictability of droplet size and nozzle blockage are further drawbacks. Finally, laser-assisted bioprinting was initially developed for metal patterning for computer chip fabrication. It consists in the printing of biological patterns to substrates using a laser as an energy source. The process involves the use of a pulsed laser source that initiates the release of bioink droplets present in a ribbon coated with the biological materials deposited on a metal film [263]. The bioink droplets are ejected onto a receiving substrate that contains a biopolymer or cell culture medium to sustain cellular adhesion and growth. This technique has a good resolution, but it has a high production cost. Also, cell death brought on by thermal damage in laser-assisted bioprinting is one of the key worries.

The choice of the bioinks’ composition is important in 3D bioprinting, since they include not only biomaterials but living cells and growth factors as well. Therefore, the components must be nontoxic and printing temperature must be lower than physiological temperatures in comparison to other conventional materials [264]. The bioinks containing decellularized ECM are known to have better regeneration capability than those containing other natural polymers, since they retain many of the previously described tissue-specific native features such as differentiation markers and proteins, promoting cell differentiation and proliferation. Given these advantages and the explosive interest in 3D bioprinting, it is not surprising the existence of commercial ECM bioinks such as Bone deCelluid™, Skin deCelluid™, and Cartilage deCelluid™ that are being considered for clinical use [[265], [266], [267]]. Indeed, decellularized ECM has been used as a bioink for several biomedical applications. For the purpose of understanding the crosstalk between mesenchymal stem cells and ECM in respiratory illnesses, Almendros and colleagues created a realistic 3D model by using cell-laden hydrogels based on lung-derived ECM as the bioink [268]. Chae and coworkers described a cell-based TE approach to produce cell-laden tissue constructs by using 3D extrusion bioprinting with mesenchymal stem cells and tissue-specific bioinks for tendon/ligament applications [269]. In cardiac TE, Das and collaborators have demonstrated the importance of the matrix and the culture microenvironment in the interactions between cells and materials when trying to establish an extrusion-based 3D-printed cardiac tissue model. In fact, cardiomyocytes presented enhanced maturation when printed in an ECM bioink, when compared with a collagen bioink [270]. Similarly, progress in this field has been observed for several other tissue types, such as liver [271], adipose [272], skin [267], bone [273], muscle [274], and kidney [275].

Notwithstanding these major recent developments, there are some limitations that persist, and improvements that need to be considered regarding this technique. Despite substantial research, it is still difficult to obtain mechanically strong tissue structures. This is mainly due to the poor mechanical, structural, and gelation properties of the bioinks produced exclusively using decellularized ECM. So, to mitigate these problems, the typical approach is to combine other biomaterials with the decellularized ECM or provide extra crosslinking agents. Other key challenges for advancing 3D bioprinting technologies include increasing structural resolution, preserving cell viability, and cutting down on manufacturing time. However, ECM-derived bioinks and 3D bioprinting still remain as promising tools to engineer tissues for TERM application.

### 5.3 Electrospinning

**Fig. 11:**
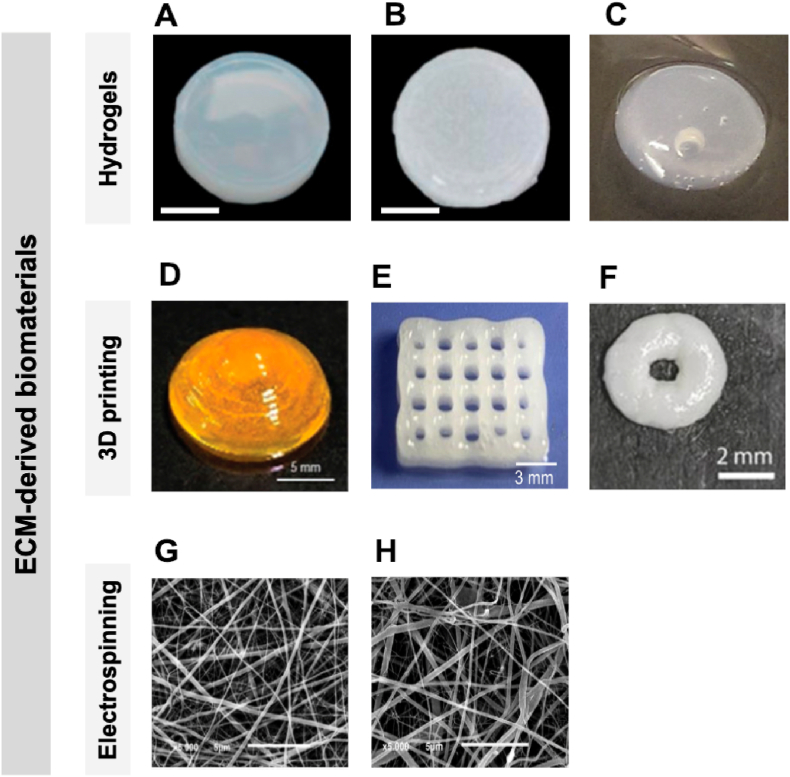
Images of ECM-derived applications of **hydrogels** (A) based on dermal ECM, (B) porcine urinary bladder matrix, scale bar = 1 cm. Adapted with permission from Ref. [292]. Copyright 2012, Elsevier; (C) human pancreatic parenchym dECM. Adapted with permission from Ref. [119]. Copyright 2018, Springer Nature. **3D bioprinted scaffolds** (D) dECM 3D printed curved cornea. Adapted with permission from Ref. [293]. Copyright 2021, John Wiley and Sons; (E) 3D bioprinted construct using cell-laden cartilage-derived methacrylate ECM bioink. Adapted with permission from Ref. [294]. Copyright 2021, Elsevier; (F) 3D printed hydrogel composed of methacrylate dECM and methacrylate PCL. Adapted with permission from Ref. [295]. Copyright 2020, Elsevier. **electrospinning** (G) SEM micrographs of electrospun blends of dECM:PCL (70:30), and (H) electrospun blends of dECM:PCL (50:50). Adapted with permission from Ref. [275]. Copyright 2019, Elsevier.

In recent years, the electrospinning technique has been used to produce ECM-derived scaffolds with oriented multifilament nanofibers that are structurally similar to the nanofibrous framework of the ECM in the native environment. Electrospinning is a manufacturing method that applies electric voltage (10–40 kV) to a metallic needle carrying a solution, such as solubilized ECM, creating a 3D fibrous network with fibers that ranges in size from nanometers to micrometers [276]. The fibers are dropped onto a collector, where they are piled to create a scaffold with the appropriate shape and architecture to help induce cell-specific functions such as adhesion and migration. These scaffolds offer nano-scale fiber structures with interlinked pores to resemble the native ECM, demonstrating excellent potential to create functional tissues [277]. However, several conditions need to be optimized to create ECM architecturally analogous configurations. The structure and diameter of the manufactured fibers are controlled by factors such as the viscosity of the solution, surface tension, and the molecular weight [278,279]. Additionally, environmental factors such as temperature and humidity also influence the fibers morphology [280]. Thus, controlling the abovementioned factors is essential for creating appropriate fibers. This technique is highly versatile, rapid, efficient and relatively inexpensive, since it only depends on a high voltage supply, a collector plate and a syringe as spinneret for the electrospinning setup [281]. As previously mentioned, decellularized ECM can be used with electrospinning technologies to create ECM-like scaffolds for TERM applications such as wound healing [282], peripheral nerve [283], cartilage [284], liver [285], neural [286], and bone regeneration [145]. Ancillary methods that improve the performance of electrospun scaffolds, like surface functionalization and emulsion-based electrospinning, have also been reported [[287], [288], [289], [290], [291]]. Fig. 11 depicts examples of the three TERM applications here described for ECM.

## 6 Final remarks

An overview of the use of ECM-derived biomaterials in the TERM field, from the isolation to the application stage, with significant emphasis on the characterization part, is herein presented. Several challenges remain for the full realization of the clinical potential of these materials.

Decellularization techniques, a key step to obtain ECM-derived biomaterials, are complex and challenging. Achieving complete cell removal while preserving ECM desired properties is the object of intense research. Most works report the use of more than one decellularization method in order to achieve the most satisfactory results. But the amalgamation of different complex methods results in significant batch-batch variability. And this variability is compounded by small changes in the protocols between different laboratories, hindering scientific progress and the clinical potential of these materials. Therefore, the standardization of decellularization protocols is something desirable, if not required, for the widespread clinical application of ECM-based biomaterials. While still an elusive goal, automated systems such as the one proposed by the Freytes lab will most likely be key tools for researchers in the field [296]. This system allows a fast, effective and reproducible decellularization process that was proven to yield biocompatible hydrogels starting from various different tissue sources.

An essential part of the effort to validate the decellularization protocols is the choice of adequate characterization techniques. And while there is in fact a large set of techniques available, a complete characterization of the ECM is very time and resource intensive. Moreover, most techniques require a pre-processing of the sample, in which important information may be lost or modified prior to analysis. Improving sample preparation protocols and equipment sensitivity are requirements to improve the characterization of isolated ECM. We believe that a wide set of characterization techniques is currently indispensable for researchers working in decellularized ECM-derived biomaterials for TERM (Fig. 5). However, the standardization of decellularization protocols will most likely allow to reduce the characterization burden, making its full definition contingent on the desired final application of the materials. In fact, when envisaging TERM applications, different challenges arise. The application specificities will determine key features of the ECM-derived product such as the type of scaffold, the processing technique, mechanical properties, degradability, biocompatibility and cytocompatibility, just to name a few. And the tuning of these features can depend on the extraction and decellularization processes. Therefore, the application requirements must be precisely determined so that the upstream processes are adjusted accordingly. But, once again, standardized protocols are the only way to efficiently implement this back-and-forth process where reproducibility is key.

One common feature of ECM-based biomaterials that may be considered limiting in the context of TERM applications is the relatively weak mechanical properties of derived scaffolds. This is a limitation usually managed after extraction and decellularization, resorting to chemical crosslinkers that endow the final construct with improved mechanical properties. The required additional processing steps can be detrimental to the biocompatibility of the final scaffold since they usually involve chemical modifications that impact properties of the biomaterials other than the mechanical. Reagents such as glutaraldehyde have excellent crosslinking efficiency but have been shown to induce cytotoxic effects *in vitro* and *in vivo* [297]. Therefore, the search for crosslinkers that are efficient without impacting the biocompatibility of the biomaterial is and will be an essential topic for the widespread application of ECM-derived biomaterials in TERM applications. The use of natural compounds derived from plants such as genipin or poliphenols as crosslinkers has shown to be promising in that regard [216,297] and therefore we believe that other natural compounds derived from earth or sea organisms [297,298] may be part of the solution for the crosslinking problem.

While all the issues described above are important hurdles, ongoing research suggests they will be solved by the community in the short to medium term. New challenges will arise with more comprehensive preclinical and clinical testing of products based on ECM-derived biomaterials. Immunogenicity issues will for sure be among those challenges [299]. We foresee that a closer interaction between clinicians and material scientists and biomedical engineers will be key to overcome them, as already acknowledged in the biomaterial field in general.

## Ethic approval

Not applicable.

## CRediT authorship contribution statement

**Helena Vilaça-Faria:** Writing – original draft, Investigation. **Jennifer Noro:** Writing – original draft, Investigation. **Rui L. Reis:** Visualization, Supervision. **Rogério P. Pirraco:** Writing – review & editing, Supervision, Funding acquisition, Conceptualization.

## Declaration of competing interest

There are no conflicts to declare.
